# A lactate-related LncRNA model for predicting prognosis, immune landscape and therapeutic response in breast cancer

**DOI:** 10.3389/fgene.2022.956246

**Published:** 2022-10-05

**Authors:** Jia Li, Yinbin Zhang, Chaofan Li, Huizi Wu, Cong Feng, Weiwei Wang, Xuan Liu, Yu Zhang, Yifan Cai, Yiwei Jia, Hao Qiao, Fei Wu, Shuqun Zhang

**Affiliations:** ^1^ Department of Oncology, The Second Affiliated Hospital of Xi’an Jiaotong University, Xi’an, China; ^2^ Department of Orthopedics, The Second Affiliated Hospital of Xi’an Jiaotong University, Xi’an, China

**Keywords:** lactate, long non-coding RNA, breast cancer, prognostic signature, tumor immune microenvironment, drug sensitivity

## Abstract

Breast cancer (BC) has the highest incidence rate of all cancers globally, with high heterogeneity. Increasing evidence shows that lactate and long non-coding RNA (lncRNA) play a critical role in tumor occurrence, maintenance, therapeutic response, and immune microenvironment. We aimed to construct a lactate-related lncRNAs prognostic signature (LRLPS) for BC patients to predict prognosis, tumor microenvironment, and treatment responses. The BC data download from the Cancer Genome Atlas (TCGA) database was the entire cohort, and it was randomly assigned to the training and test cohorts at a 1:1 ratio. Difference analysis and Pearson correlation analysis identified 196 differentially expressed lactate-related lncRNAs (LRLs). The univariate Cox regression analysis, least absolute shrinkage and selection operator (LASSO), and multivariate Cox regression analysis were used to construct the LRLPS, which consisted of 7 LRLs. Patients could be assigned into high-risk and low-risk groups based on the medium-risk sore in the training cohort. Then, we performed the Kaplan–Meier survival analysis, time-dependent receiver operating characteristic (ROC) curves, and univariate and multivariate analyses. The results indicated that the prognosis prediction ability of the LRLPS was excellent, robust, and independent. Furthermore, a nomogram was constructed based on the LRLPS risk score and clinical factors to predict the 3-, 5-, and 10-year survival probability. The GO/KEGG and GSEA indicated that immune-related pathways differed between the two-risk group. CIBERSORT, ESTIMATE, Tumor Immune Dysfunction and Exclusion (TIDE), and Immunophenoscore (IPS) showed that low-risk patients had higher levels of immune infiltration and better immunotherapeutic response. The pRRophetic and CellMiner databases indicated that many common chemotherapeutic drugs were more effective for low-risk patients. In conclusion, we developed a novel LRLPS for BC that could predict the prognosis, immune landscape, and treatment response.

## Introduction

Breast cancer (BC) is the most common tumor and ranks fifth in cancer-related death globally (Harbeck and Gnant, 2017). Although early detection, diagnosis, and treatment for BC have made significant progress, cancer recurrence, distant metastasis, and drug resistance are still prevalent in patients with BC (Jabbarzadeh kaboli et al., 2020). Many stratification terms have been built for the precise treatment of diseases, and polygenic makers may be more accurate than conventional methods (Li et al., 2019). BC is most commonly classified into five subtypes using PAM50, including luminal A, luminal B, HER2-enriched, normal-like, and basal-like (Harbeck et al., 2019). However, the considerably heterogeneous nature of tumors limits the broad applicability of typing (Waks and Winer, 2019). It is essential to investigate new potential markers for prognostic prediction and provide patients personalized treatments.

Lactate is the endpoint of anaerobic glycolysis and usually is considered an endpoint or waste metabolite in cancer. Recent studies indicate that lactate is an essential regulator of cancer development, maintenance, tumor microenvironment, and metastasis (Doherty and Cleveland, 2013; Hayes et al., 2021). In breast cancer, GPR81 is upregulated and promotes tumor growth by releasing lactate from tumor cells (Longhitano et al., 2022). Lactate dehydrogenase A might be a prognostic marker in clear cell renal cell carcinoma (Girgis et al., 2014). Lactate/BDNF/TrkB signaling could mediate epithelial-stroma interaction and lead to anlotinib resistance in gastric cancer (Jin et al., 2021). In addition, lactate takes part in epigenetic regulation. Histone lysine lactylation is involved in regulating gene transcription (Izzo and Wellen, 2019).

Numerous studies have demonstrated that lactate is relevant to the tumor immune microenvironment (TIME) and immunotherapy. Elevated lactate levels are the primary cause of tumor microenvironment (TME) acidosis, suppressing CD8 + and CD4 + effector T cell function, and favoring immunosuppressive Treg development (Nakagawa et al., 2015; Corbet and Feron, 2017; Erra díaz et al., 2018). As to Innate immunity, tumor-associated macrophages (TAMs) could subvert anti-tumor immune responses and act as a negative prognostic marker (Gabrilovich et al., 2012). Lactate could promote transcriptional polarization of TAM towards the tumor-promoting M2 phenotype in cervical (Stone et al., 2019), breast (Mu et al., 2018), lung cancer and melanoma (Zhang et al., 2019a). In addition to surgery, chemotherapy, radiotherapy and targeted therapies, immunotherapy is the fifth element of cancer treatment. However, the immunosuppressive heavy tumor microenvironment often limits immunotherapy and other therapeutic efficacy. Studies have found that elevated lactate levels can affect the therapeutic efficacy and overall survival of immune checkpoint inhibitors for melanoma (Kelderman et al., 2014), esophageal squamous cell carcinoma (Wang et al., 2019), and non-small cell lung cancer (Zhang et al., 2019b).

LncRNA consists of RNA molecules with at least 200 base pairs that originate from the non-coding region of the genome, involved in almost all human biological processes and series of diseases (Esteller, 2011; Hauptman and Glavač, 2013; Fatica and Bozzoni, 2014). Several studies have reported that lncRNAs could regulate lactate metabolism and immune status in different cancers. The lncRNA SNHG5 regulates BACH1 *via* miR-299 to promote glycolysis and proliferation in breast cancer cells (Huang et al., 2022). LncRNA NEAT1-associated aerobic glycolysis in prostate cancer could blunt tumor immunosurveillance by T cells (Xia et al., 2022). Furthermore, lncRNAs could be used as novel immunotherapeutic tools against cancer, and immunotherapy based on lncRNAs could increase the effectiveness and reduce off-target effects (Kaur et al., 2022). Together, lncRNA plays a role in diagnosing, prognosis, and treating BC (Rodríguez bautista et al., 2018).

Research has shown the critical value of lactate and lncRNAs in cancer classification, prognosis, and immunotherapy (Sun et al., 2022; Xie et al., 2022; Xu et al., 2022). However, lactate-related lncRNAs have not been well studied in BC. Our study developed and verified an LRLPS to predict BC patients’ prognosis, immune infiltration and therapeutic response by applying bioinformatics. As a result, our findings may provide new clues for cancer prognosis evaluation and treatment guidance.

## Materials and methods

### Data collection

The R package “TCGAbiolinks” was used to acquire transcriptome profiling, simple nucleotide variations, and the clinical information of TCGA-BRCA patients (Colaprico et al., 2016). We excluded male patients and retained 1096 BC and 112 normal samples for the differential analysis. Furthermore, 916 BC samples with the OS > 30 days were included in the prognostic analysis. They were randomly divided into the training (*n* = 458) and test (*n* = 458) cohorts at a 1:1 ratio using the “caret” R package. Clinical characteristics of the three cohorts were analyzed with the “tableone” R package (Suppplementary Table S1). In the subsequent clinicopathological correlation analysis, we excluded patients with incomplete information. We acquired 284 lactate-related genes by querying the Molecular Signatures Database with “lactic” as the search keyword (Suppplementary Table S2) (Liberzon et al., 2015).

### Identification of differential expressed lactate-related lncRNAs in Breast cancer

The “EdgeR” R package assessed the differentially expressed lncRNAs and lactate-related genes (*p* < 0.05, |log2FC| = 1). For further study, we retained differential expression lncRNAs expressed in more than half of the patients. Further identification of lactate-related lncRNAs was performed with Pearson correlation analysis at a standard of |R| > 0.4 and the *p*-value < 0.001.

### Construction and validation of the lactate-related lncRNAs prognostic signature

The univariate Cox regression analysis identified the prognostic LRLs in the training cohort. We performed LASSO with the R package “glmnet” to avoid overfitting (Friedman et al., 2010). Then, the LRLPS was built with the multivariate Cox regression analysis based on the stepwise Akaike information criterion (stepAIC) value. According to the LRLPS, each sample could get the risk score with the following formula: Risk score = *Σ*(Exp∗ Coef). The Coef and Exp were the coefficients and the expression level of each lncRNA, respectively. The high- and low-risk groups were divided according to the median risk score of the training cohort. We further performed the Kaplan–Meier survival analysis, time-dependent ROC curves, and univariate and multivariate analyses to evaluate the accuracy and independence of the LRLPS in prognosis prediction in the three cohorts.

### Stratified analysis and construction of the nomogram

The stratified analysis could assess the prognosis value of LRLPS in different subgroups stratified by several clinical features, including age, pathologic stage, T stage, N stage, M stage, ER, PR, and HER2 statuses. We constructed the nomogram with the independent prognostic factors. Nomogram accuracy was evaluated through ROC curves, C-index, and calibration curves. Finally, we measured the net benefit of using a nomogram and other clinical features alone based on decision curve analysis (DCA).

### Functional enrichment analysis

We identified the differentially expressed genes (DEGs) between the two risk groups and annotated their functions with Gene Ontology (GO) and Kyoto Encyclopedia of Genes and Genomes (KEGG) using the R package “ClusterProfiler” (Yu et al., 2012). The variations of pathway activity of the subgroups were further revealed with Gene Set Enrichment Analysis (GSEA) (*p* < 0.05 and FDR<0.25) (Subramanian et al., 2007). Annotated gene set “c2. cp.kegg.v7.5.1.symbols.gmt” could acquire from the MSigDB (https://www.gsea-msigdb.org/gsea/msigdb/).

### Evaluation of immune infiltration and immunotherapy response in the two risk groups

We evaluated the proportion of tumor-infiltrating immune cells through the CIBERSORT algorithm (Newman et al., 2015). Tumor purity, immune, stromal, and estimate scores were evaluated through the ESTIMATE algorithm (Yoshihara et al., 2013). Furthermore, we assessed twenty-seven potential immune checkpoints (ICPs) in the two risk groups. In order to predict immune checkpoint inhibitor (ICI) responses, we applied IPS and TIDE. TIDE was an online analysis that could predict the response to ICIs (http://tide.dfci.harvard.edu/) (Jiang et al., 2018; Fu et al., 2020). The IPS is a machine learning-based system, scored as z scores according to four immunogenicity-related cell types (effector cells, immunosuppressive cells, MHC molecules, and immunomodulators), and it was positively correlated with immunogenicity (Charoentong et al., 2017). It is reported that IPS could assess the tumor immunogenicity and response to ICI therapy in various tumor types. The IPS of BC patients were downloaded from The Cancer Immunome Atlas (TCIA) (https://tcia.at/home).

### Correlation between the risk score and tumor mutation

The mutation landscapes in the two risk groups were analyzed with the “maftools” R package. Mutations in the genome per million bases are known as the tumor mutational burden (TMB), a potential immunotherapy biomarker (Lu et al., 2019; Shum et al., 2022). We evaluated the TMB in the two risk groups and explored the association between TMB and the risk score.

### Evaluation of the drug sensitivity and potential target drugs

The “pRRophetic” R package was used to calculate the half-maximal inhibitory concentrations (IC50) of the common chemotherapy drugs based on the Genomics of Drug Sensitivity in Cancer (GDSC; https://www.cancerrxgene.org/) database (Yang et al., 2013; Geeleher et al., 2014). As to the lncRNAs in the LRLPS, we explored the potential target drugs (approved by the FDA and those in clinical tests) with the CellMiner database (https://discover.nci.nih.gov/cellminer) (Shankavaram et al., 2007; Shankavaram et al., 2009). The relationship between model lncRNAs and drug sensitivity was studied using Pearson correlation analysis.

### Statistical analysis

We applied R software (version 4.0.5, https://www.r‐project.org/) for all statistical analyses. *p*-value < 0.05 was set as statistically significant, and the significance levels were set as *p ≤ 0.05, **p ≤ 0.01, ***p ≤ 0.001 and ns = *p* > 0.05.

## Results

### Identification of the differentially expressed lactate-related lncRNAs in Breast cancer patients

**FIGURE 1:**
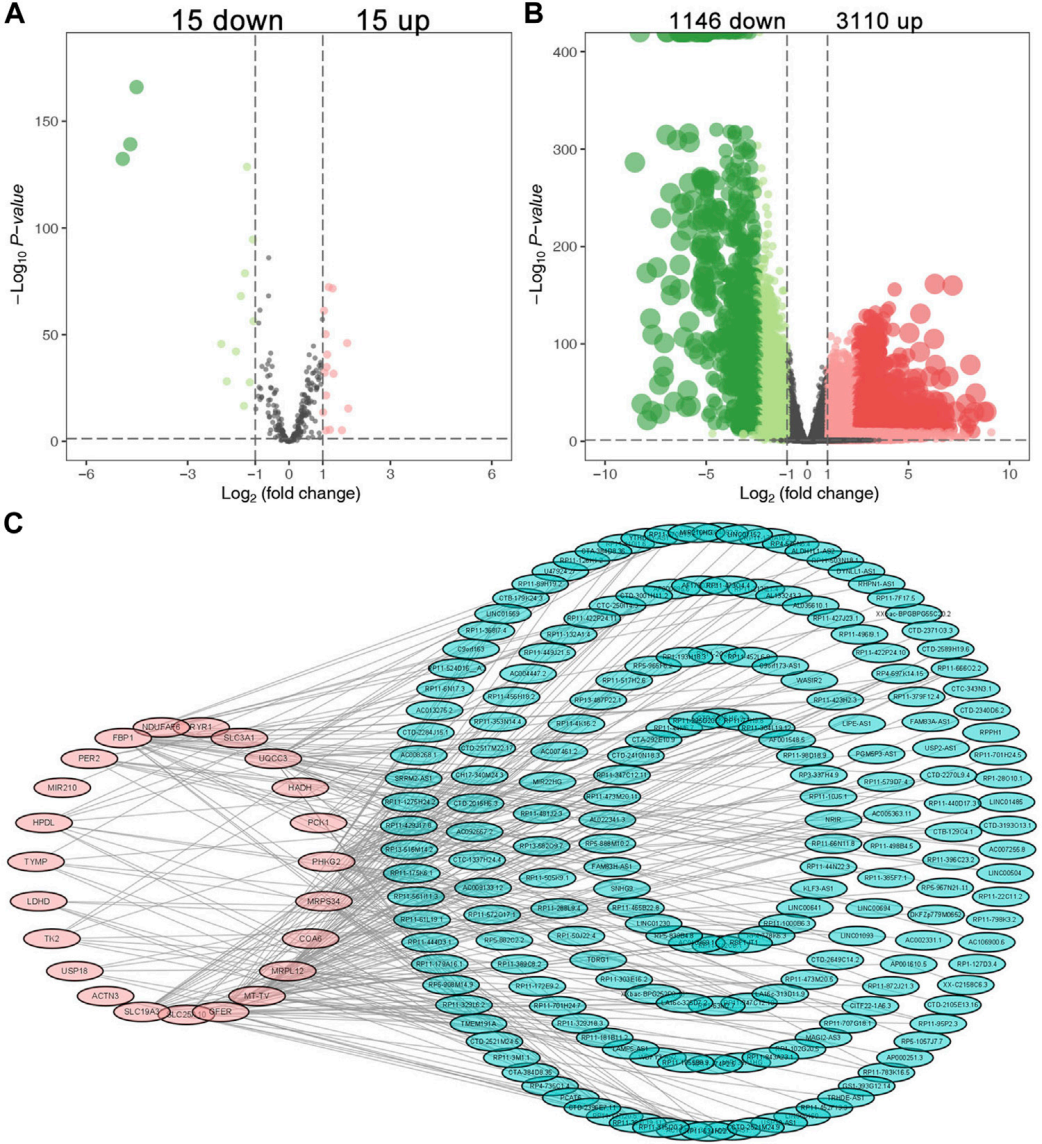
Identification of the differential expressed LRLs. The volcano plots of the differentially expressed lactate-related genes **(A)** and differentially expressed lncRNAs **(B)**. **(C)** The interaction between the differentially expressed lactate-related genes and LRLs.

There were 30 differentially expressed lactate-related genes and 4,256 differentially expressed lncRNAs, respectively (Figures 1A,B). Based on Pearson correlation analysis, we identified 196 differentially expressed LRLs for further investigation. Figure 1C showed the interaction between the lactate-related genes and LRLs.

### Development and evaluation of the lactate-related lncRNAs prognostic signature

**FIGURE 2:**
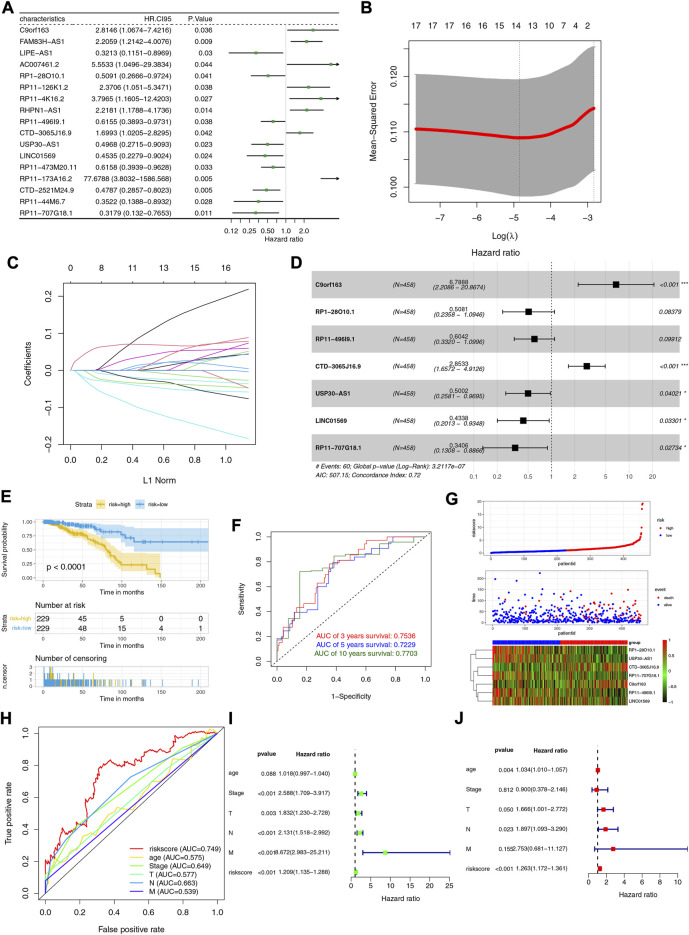
Construction and evaluation of the LRLPS. **(A)** The univariate Cox regression analysis of LRLs in the training cohort. **(B)** The cross-validation graph shows the optimal parameter selection with minimum criteria in the LASSO model. **(C)**The LASSO coefficient profiles of the 14 LRLs. **(D)** The forest graph showed the results of stepwise multivariable cox proportional hazards regression analysis. **(E)** The OS curve of the two risk groups. **(F)** The time-dependent ROC curves of the LRLPS. **(G)** The risk score, clinical event, and the model genes in the two risk groups. **(H)** The ROC curves of the risk score and other clinicopathological parameters. The univariate **(I)** and multivariate **(J)** Cox regression analyses.

In the training cohort, 17 LRLs with prognostic values were identified with the univariate Cox regression analysis (Figure 2A). We performed LASSO cox analysis and identified 14 LRLs to avoid overfitting the model (Figures 2B,C). The multivariate Cox regression analysis identified 7 LRLs to construct the LRLPS based on the lowest AIC 507.17 (Figure 2D). Each patient would acquire a risk score by calculating the following: risk score= (1.915272666 *C9orf163) + (−0.677100153 * RP1-28O10.1) + (−0.503780886 * RP11-496I9.1) + (1.048467864 * CTD-3065J16.9) + (−0.692769124 * USP30-AS1) + (−0.835154753 * LINC01569) + (−1.077081426 * RP11-707G18.1) (Supplementary Table S3). Subsequently, we evaluated the ability of the LRLPS in prognosis prediction. Kaplan-Meier analysis showed that patients in the high-risk group had shorter overall survival (OS) (Figure 2E). The area under the 3-, 5-, and 10-year time-dependent ROC curves (AUC) were 0.7536, 0.7229, and 0.7703, respectively, which indicated the accuracy of the LRLPS (Figure 2F). Figure 2G indicated the correlation between the risk score and the outcome of BC patients. Figure 2H indicated that the AUC of risk score was the highest (0.749), followed by the N stage (0.663) and pathological stage (0.649). The univariate (Figure 2I) and multivariate (Figure 2J) Cox regression analyses indicated the independent prognostic value of the risk score.

### Validation of the lactate-related lncRNAs prognostic signature

**FIGURE 3:**
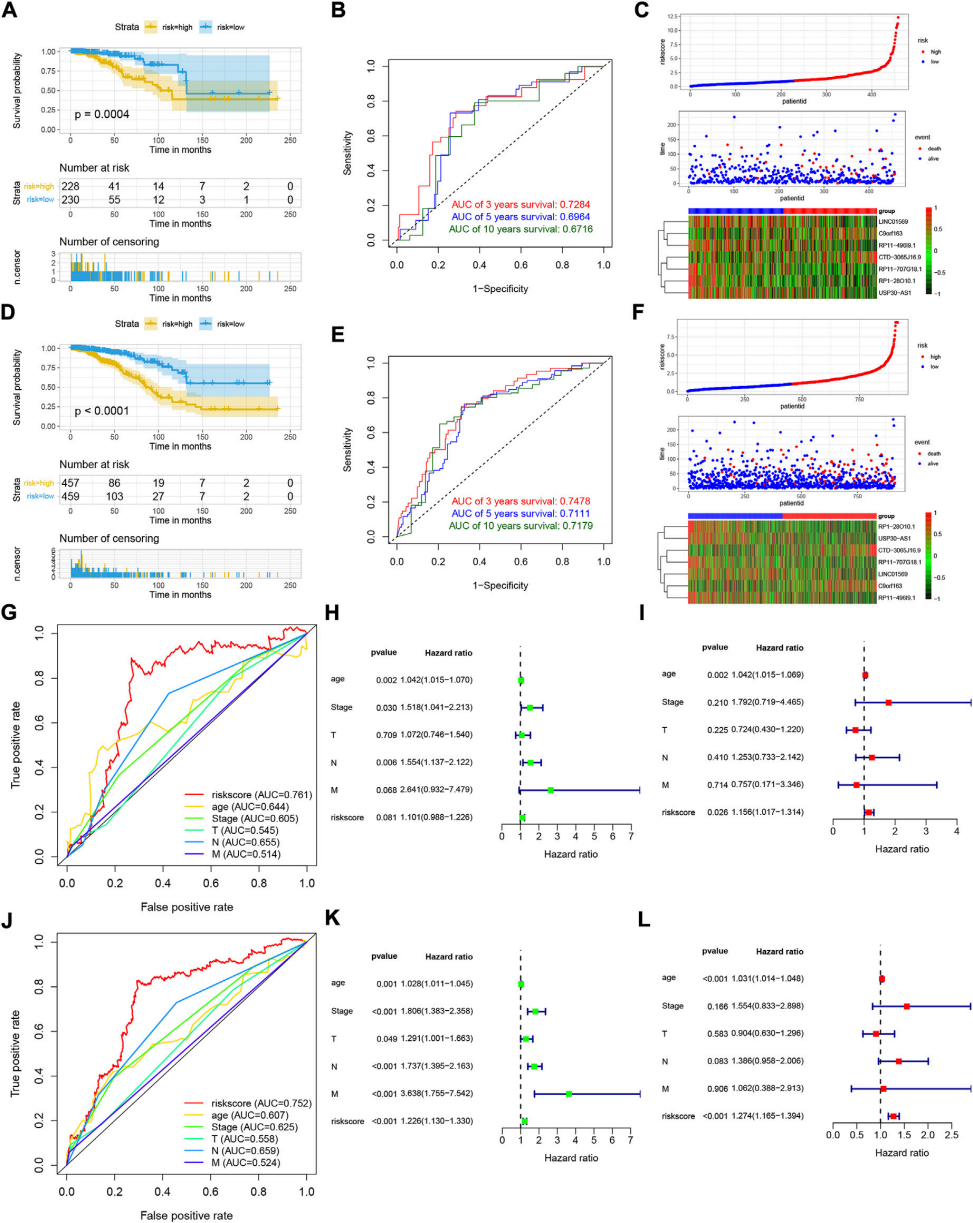
Validation of the LRLPS. The OS curve of the two risk groups in test **(A)** and entire **(D)** cohorts. The time-dependent ROC curves in test **(B)** and entire **(E)** cohorts. The risk score, clinical event, and the model genes in the two risk groups in test **(C)** and entire **(F)** cohorts. The ROC curves of the risk score and other clinicopathological parameters in test **(G)** and entire **(J)** cohorts. The univariate Cox regression analyses in the test **(H)** and entire **(K)** cohorts. The multivariate Cox regression analyses in the test **(I)** and entire **(L)** cohorts.

**FIGURE 4:**
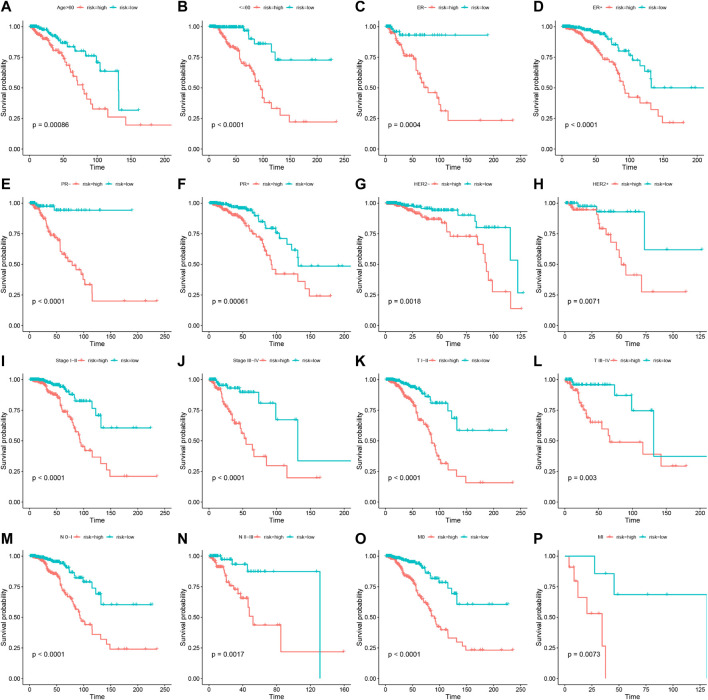
Stratification analyses of the prognostic signature. Kaplan-Meier curves indicated the OS of the two risk groups stratified by age (>60 years vs. ≤60 years) **(A,B)**, ER stage (negative vs. positive) **(C,D)**, HER2 stage (negative vs. positive) **(E,F)**, PR stage (negative vs. positive) **(G,H)**, stages (stage I–II vs. stage III-IV) **(I,J)**, AJCC T stage (T I–II vs. T III-IV) **(K,L)**, AJCC N stage (N 0-I vs. T II-III) **(M,N)**, AJCC M stage (M 0 vs. M I) **(O,P)**, respectively.

To assess the stability of the LRLPS, we used the same analyses in the test and entire cohorts. High-risk patients always had a worse OS than low-risk patients in the two cohorts (Figures 3A,D). The AUCs of the 3-, 5-, and 10-year ROC curves were 0.7284, 0.6964, and 0.6716 in the test cohort (Figure 3B), and 0.7484, 0.7111, 0.7179 in the entire cohort (Figure 3E). Figures 3C,F indicated that the higher risk score was correlated with increased mortality. In the test cohort, the AUC of the risk score was the highest (0.761) (Figure 3G). The risk score was an independent prognostic factor in the multivariate Cox regression analysis but not a statistically significant independent prognostic according to the univariate Cox regression analysis (Figures 3H,I). In the entire cohort, the risk score had the highest AUC (0.752) and was an independent prognostic factor (Figure 3J-L). We performed the stratification and Kaplan-Meier survival analyses to further explore whether the signature was suitable for different clinical subgroups. There were always significant differences in survival between the two risk groups in all clinical subgroups (Figures 4A–P). The results indicated that the prognostic signature was accurate, independent, and widely applicable.

### Comparison with other prognostic signatures

The robustness of our LRLPS was assessed by comparing it with 11 existing OS‐related lncRNAs prognostic signatures, such as ferroptosis, pyroptosis, necroptosis, N6-methyladenosine, CD4 + conventional T cells, immunity, and autophagy. We included only signatures from the TCGA database to eliminate the effects of heterogeneity. Signatures were analyzed based on their AUC, with larger AUCs showing better classification ability (Fawcett, 2006). As shown in Table 1, we have integrated all the important information of the eleven signatures, including the author, year, gene signature, and the AUCs for the signatures (Table 1). Our signature had many advantages in predicting OS in BC patients. In our study, the AUCs of the signatures at 3-, 5-, and 10-year were 0.7536, 0.7229, and 7,703, respectively, significantly higher than most hallmark predictive models. Table 1 showed that the 3-, 5-, and 10-year AUCs of another 7 lncRNA prognostic signatures, namely, the ferroptosis- and Immune-related lncRNA signature (3-, 5-, and 10-year AUCs: 0.71, 0.63, and 0.68) (Wei et al., 2022) and necroptosis-related lncRNA signature (3-, 5-, and 10-year AUCs: 0.643, 0.641, and 0.694) (Chen et al., 2022) had lower AUCs than ours; while the 12 hypoxia-related lncRNA signature (3-, 5-, and 10-year AUCs: 0.727, 741, and 0.786) (Gu et al., 2021) were comparable to the predictive capabilities of our predictive model, and our signature stand out with a clear advantage in predicting the short-term survival of BC patients. We also listed the other signatures that focus more on short-term (3- and 5-year survival) survival. We found our signature usually have better short-term survival prognostic value compared with them, such as the necroptosis-related lncRNA signature (Zhang et al., 2022), hypoxia-related lncRNA signature (Zhao et al., 2021), autophagy-related lncRNA signature (Li et al., 2021), ferroptosis-related lncRNA signature (Jia et al., 2021), N6-methyladenosine-related lncRNA signature (Lv et al., 2021), acid metabolism-related lncRNA signature (Dai et al., 2022), and CD4 + conventional T cells-related lncRNA signature (Ning et al., 2022) and pyroptosis-related lncRNA signature (Yang et al., 2021). In addition, our model only involves 7 lncRNAs, while other models (6/11) tend to have more, which is more convenient to use to a certain extent. The results indicated that our gene signature predicted BC prognosis better than most other signatures.

**TABLE 1:** The area under the ROC curve (AUC) showed the sensitivity and specificity of the known gene signatures in predicting the prognosis of BC patients.

Author	Year	Gene signature	Gene number	AUC for OS
Our study	2022	Lactate	7	0.7536 (3-year), 0.7229 (5-year), 0.7703 (10-year)
Chen F, et al.	2022	Necroptosis	7	0.731 (1-year), 0.643 (3-year), 0.641 (5-year), 0.694 (10-year)
Wei T, et al.	2022	Ferroptosis and Immune	7	0.75 (1-year), 0.71 (3-year), 0.63 (5-year), 0.68 (10-year)
Gu P, et al.	2022	Hypoxia	12	0.734 (1-year), 0.727(3-year), 0.741 (5-year), 0.786 (10-year)
Zhang Y, et al.	2022	Necroptosis	4	0.696 (3-year), 0.705 (5-year), 0.664 (7-year)
Zhao Y, et al.	2021	Hypoxia	4	0.650 (1-year), 0.681 (3-year), 0.691 (5-year), 0.642 (7-year)
Li X, et al.	2021	Autophagy	18	0.724 (3-year), 0.685 (5-year)
Jia C, et al.	2021	Ferroptosis	11	0.682 (1-year), 0.710 (3-year), 0.712 (5-year)
Lv W, et al.	2021	N6-methyladenosine	6	0.677 (1-year), 0.678 (3-year), 0.692 (5-year)
Dai Y, et al.	2022	Acid Metabolism	8	0.881 (1-year), 0.766 (3-year), 0.713 (5-year)
Ning S, et al.	2022	CD4 + Conventional T Cells	16	0.742 (1-year), 0.751 (3-year), 0.723 (5-year)
Yang X, et al.	2022	Pyroptosis	10	0.75 (1-year), 0.73 (3-year), 0.73 (5-year)

### Construction and evaluation of the nomogram

**FIGURE 5:**
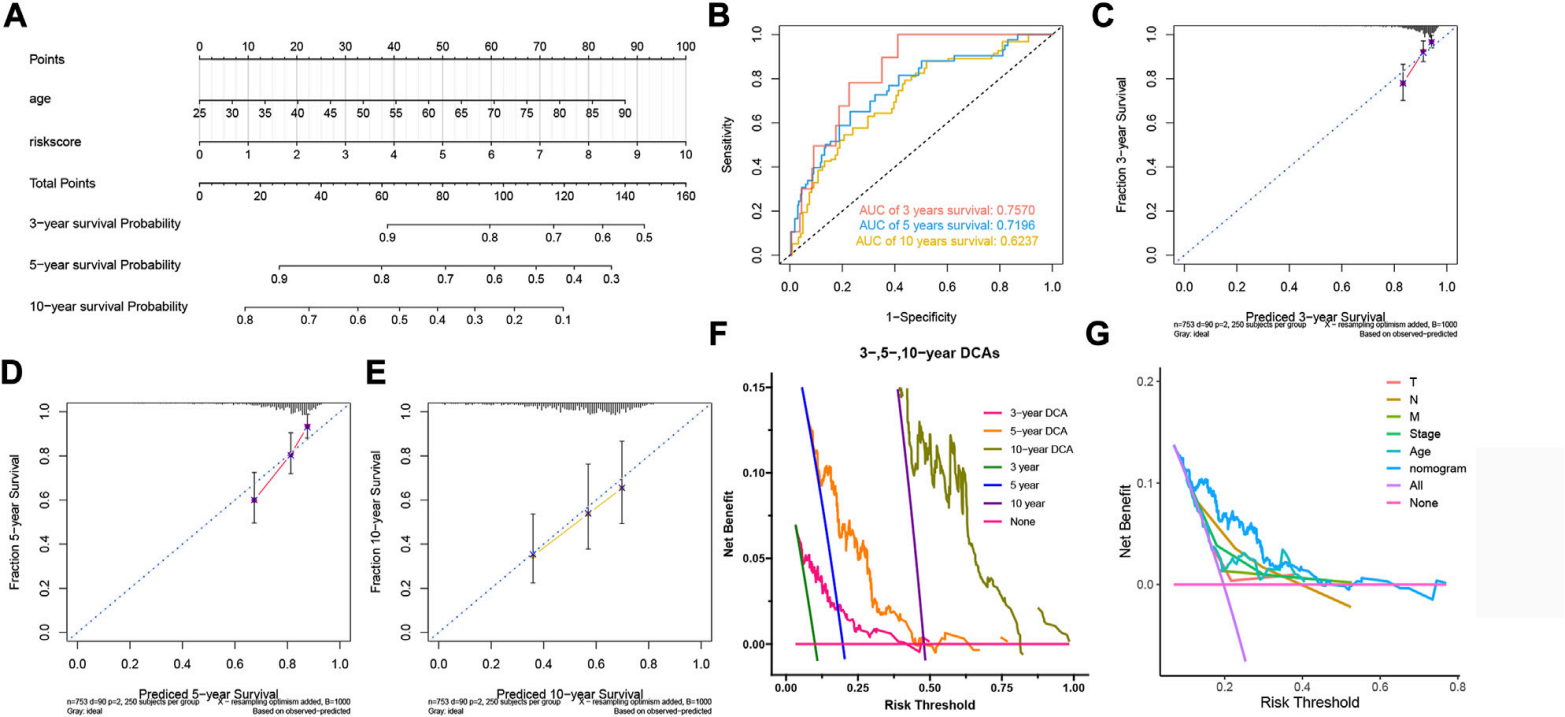
Construction and evaluation of the nomogram. **(A)** The nomogram for predicting BC patients’ survival probability. **(B)** The nomogram’s 3-, 5-, and 10-year ROC curves. **(C,D,E)** The 3-, 5-, and 10-year calibration curves. **(F)** The 3-, 5- and 10-year DCA curves of the nomogram. **(G)** DCA curves of clinicopathological factors and the nomogram.

In order to make our model better assist clinical decision-making, we constructed a nomogram that could predict the 3-, 5-, and 10-year survival probability (Figure 5A). The nomogram’s 3-, 5- and 10-year AUCs were 0.7570, 0.7196 and 0.6237, indicating the reliability of the nomogram (Figure 5B). The calibration curves proved that our prognostic nomogram could accurately predict the survival probabilities (Figures 5C–E). Furthermore, DCA curves indicated that the nomogram was more beneficial to BC patients than other clinicopathological factors (Figures 5F,G).

### Function analyses

**FIGURE 6:**
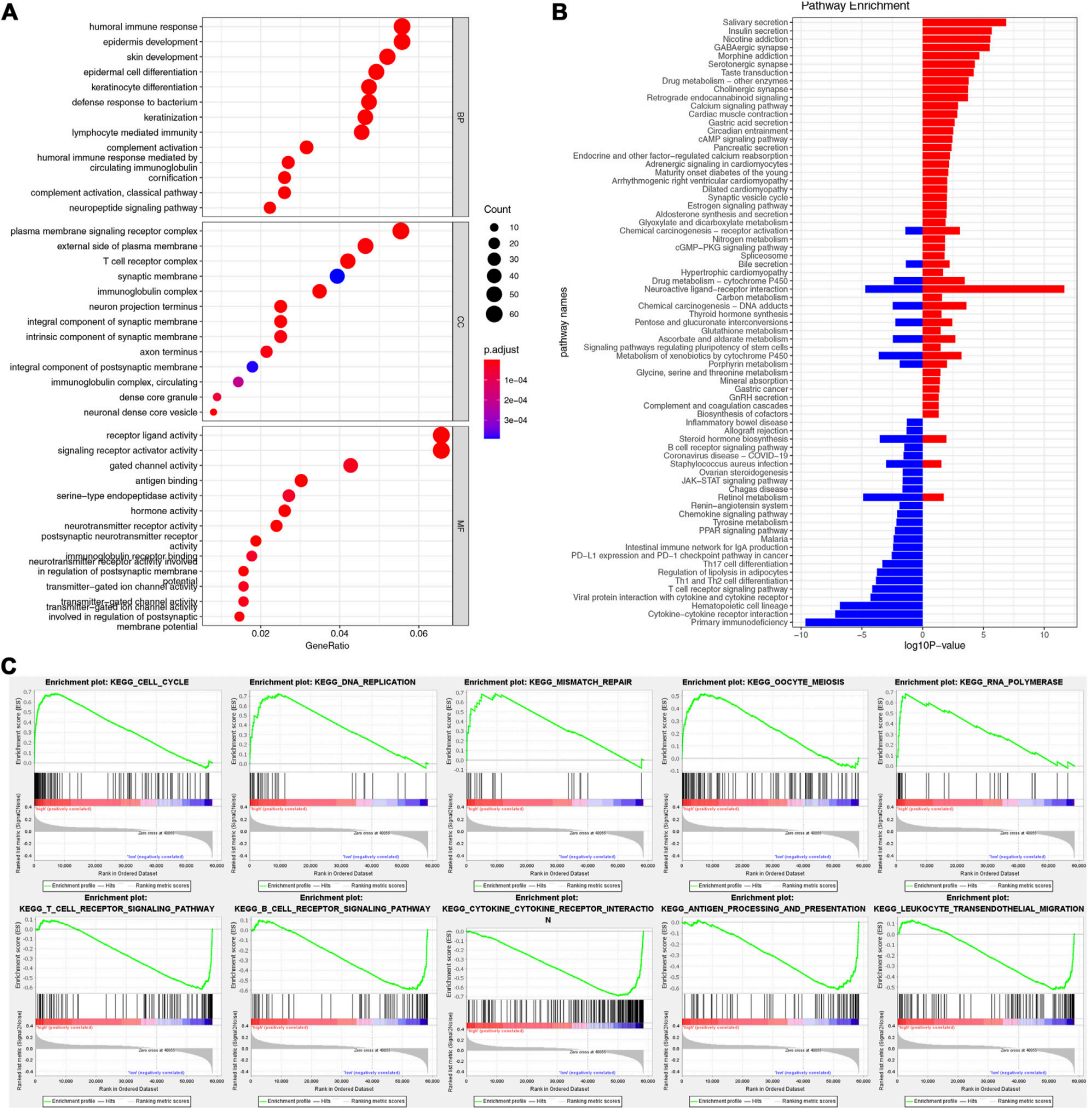
Functional enrichment analysis. **(A)** GO enrichment analysis. **(B)** KEGG enrichment analysis. **(C)** The results of GSEA in two risk groups.

We used GO/KEGG and GSEA analyses to analyze the functions of the two risk groups. There were 3,962 DEGs between the two-risk groups, including 1,524 up-regulated genes and 2,168 down-regulated genes for the high-risk group. GO analysis showed that these DEGs participated in many biological processes, such as humoral immune response, lymphocyte-mediated immunity, and epidermis development (Figure 6A). They could act as structural constituents in the T cell receptor complex, plasma membrane signaling receptor complex, and immunoglobulin complex and play an essential part in receptor ligand activity, signaling receptor activator activity, and gated channel activity (Figure 6A). KEGG analysis showed that the down-regulated genes were related to PD-L1 expression, primary immunodeficiency, cytokine-cytokine receptor interaction, and PD-1 checkpoint pathway in cancer (Figure 6B). Through GSEA analysis, we further observed the variations of pathway activity between the two risk groups. The low-risk group was enriched with the classical immune-related pathways, such as T/B cell receptor signaling pathways, leukocyte transendothelial migration, and antigen processing and presentation, while the high-risk group was enriched with the cell cycle-related pathways, including cell cycle, DNA replication, and mismatch repair (Figure 6C).

### Differential immune infiltration and immunotherapy response in the two groups

**FIGURE 7:**
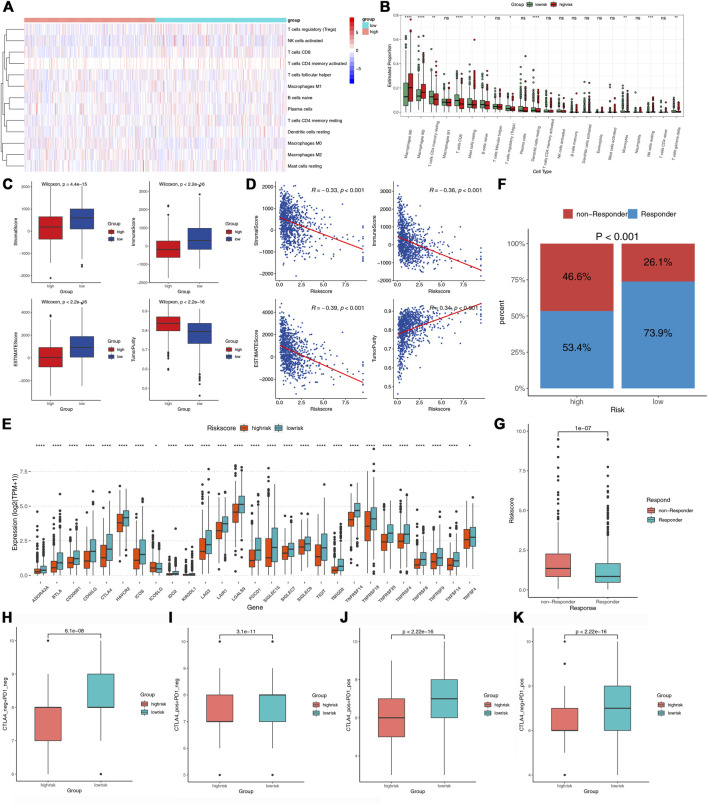
The immune infiltration and immunotherapy response in the two groups. **(A,B)** The heatmap and box plots of the proportions of tumor-infiltrating cells in the two risk groups. **(C)** Comparisons of tumor purity stromal, immune, and ESTIMATE scores between the two risk groups. **(D)** Correlations between the risk score and tumor purity, stromal, immune, and estimate score. **(E)** Comparisons of the 27 ICPs in the two risk groups. **(F)** Comparisons of the proportions of non-responders and responders to ICIs between the two risk groups. **(G)** Comparison of the risk score between the responders and non-responders. **(H–K)** Comparison of the IPS between the two risk groups.

To further study the immune landscape, we performed CIBERSORT and ESTIMATE algorithms. The heat map demonstrated the levels of the immune infiltrating cells in the two risk groups (Figure 7A). The macrophages M0, M2, and NK cells resting were the main components in the high-risk group; However, the resting CD4 T memory cells, CD8 T cells, naive B cells, activated dendritic cells, monocytes, and gamma delta T cells were mainly in the low-risk group (Figure 7B). The ESTIMATE results showed that high-risk patients had lower stromal and immune scores but had higher tumor purity (Figure 7C). Furthermore, the risk score was negatively associated with the stromal and immune scores while positively associated with tumor purity (Figure 7D). ICP was proved related to immunotherapy (Topalian et al., 2015). We assessed the expression levels of 27 ICPs in the two risk groups. They all expressed much higher in the low-risk group, such as CTLA4, HAVCR2, TIGIT, PDCD1, and LAG3 (Figure 7E). TIDE could identify the patients’ response to ICIs. As shown in Figure 7F, the low-risk group had a significantly higher response rate to immunotherapy. The risk score for non-responders to immunotherapy tended to be much higher than that for responders (Figure 7G). Furthermore, all four types of IPS were higher in the low-risk group, indicating that the low-risk patients could acquire more benefits from ICIs (Figures 7H–K). These results indicated that the low-risk group with the immune signature might have a better immunotherapy response.

In addition, we investigated whether the seven LRLs in our signature were associated with the immune signature. USP30-AS1 was significantly positively related to activated CD4 memory T cells, Macrophages M1 and CD8 T cells, and classic ICPs, such as PD-1 and CTLA4 (Supplementary Figures S1A,B). These results indicated that USP30-AS1 might make a difference in the TIME.

### Somatic mutation analysis

**FIGURE 8:**
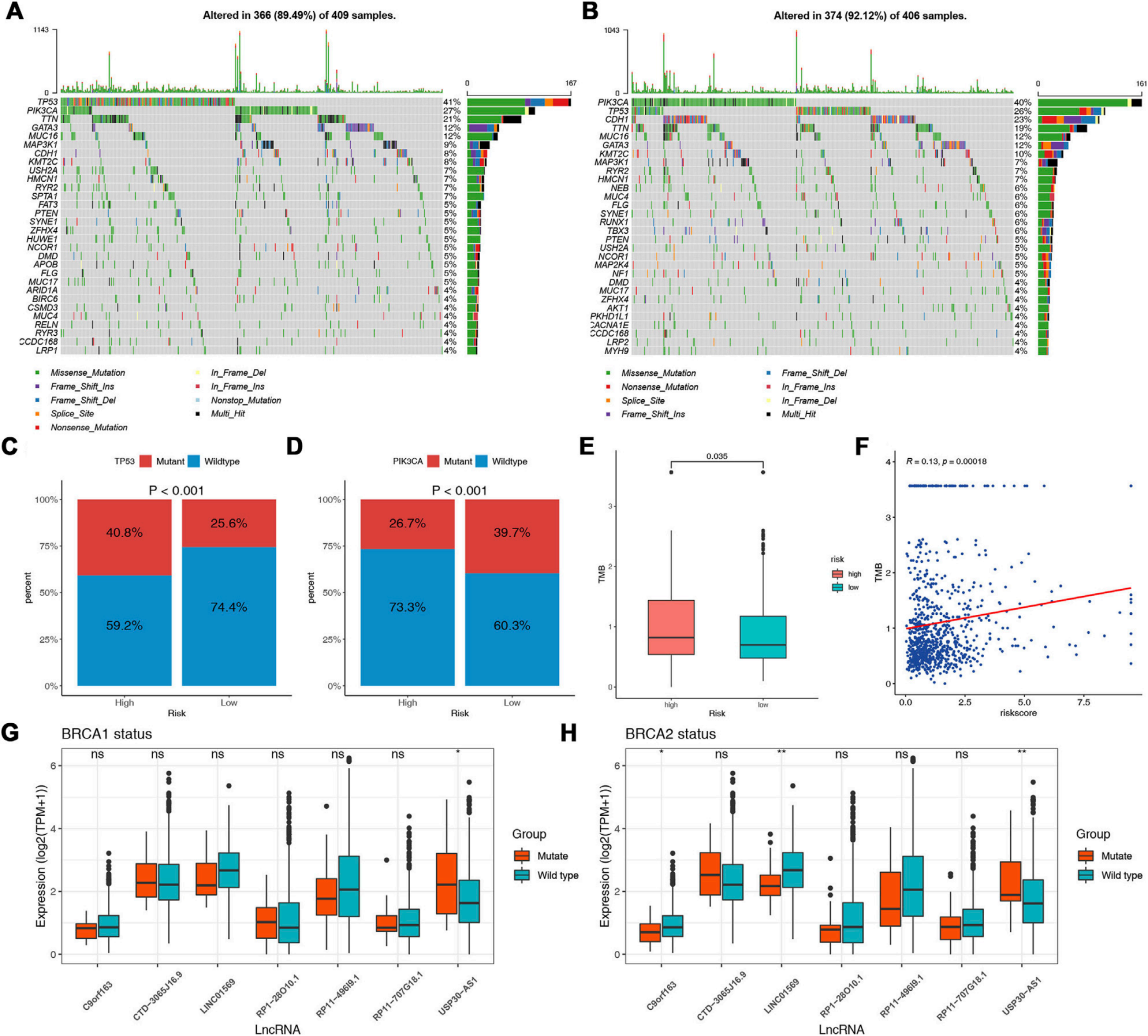
Association between DNA mutation and prognostic model. Waterfall plots of the top 30 mutated genes in the high-risk **(A)** and low-risk **(B)** groups. Comparisons of the mutation status of TP53 **(C)** and PIK3CA **(D)** in different risk groups. **(E)** Comparisons of the TMB between the two risk groups. **(F)** Correlation between TMB and the risk score. **(G)** Comparisons of the expression of the lncRNAs in different BRCA1 mutation statuses. **(H)** Comparisons of the expression of the lncRNAs in different BRCA2 mutation statuses.

The potential contribution of genomic changes to tumor immunity and immune infiltration has been explored in previous studies (Rooney et al., 2015; Thorsson et al., 2018). Figures 8A,B showed the top 30 genes mutated most frequently in the two risk groups. Although the overall mutation frequency is similar between the two groups (high vs. low, 89.49% vs. 92.12%), about one-third of the genes are different. SPTA1, APOB, ARID1A, BIRC6, GSMD3, RELN, RYR3, LRP1, and HUWE1 were not observed in the low-risk group. Regarding the most common BRCA biomarkers, the high-risk group had significantly more patients with TP53 mutations (low vs. high, 25.6 vs. 40.8%) (Figure 8C). A higher mutation frequency of PIK3CA was observed in the low-risk group (low vs. high, 39.7 vs. 26.7%) (Figure 8D). Further, the TMB in the high-risk group was significantly higher (*p* = 0.035) (Figure 8E). The Pearson correlation analysis indicated a positive correlation between TMB and the risk score (Figure 8F).

Mutations in the BRCA1 and BRCA2 genes are known risk factors and drivers of breast cancer (Kuchenbaecker et al., 2017). As to the lncRNAs identified as a molecular signature in our analysis, we further explore the associations between the expression of the lncRNAs and BRCA1/2 mutation status. We found that the expression of USP30-AS1 was significantly increased in the BRCA1/2 mutant group, and in addition, C9orf163 and LINC01569 were significantly decreased in the BRCA2 mutant group (Figures 8G,H). We did not find an association between the expression of other lncRNAs and BRCA1/2 mutation status.

### Prediction of potential drugs and the sensitivity of chemotherapeutic agents

**FIGURE 9:**
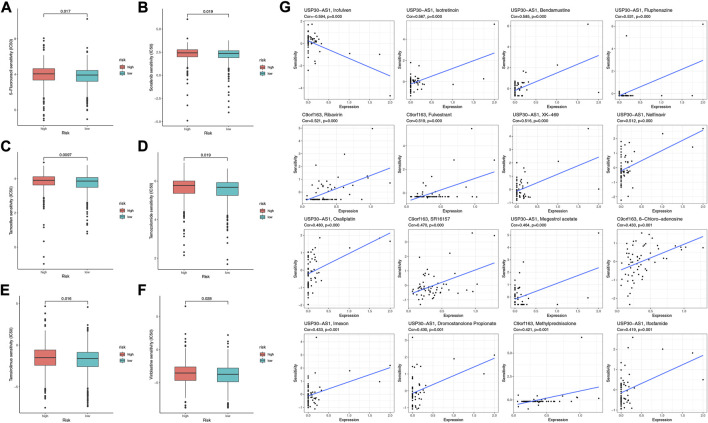
The sensitivity of chemotherapeutic agents and the prediction of potential drugs. The IC50 values of six chemotherapy and targeted agents in the two risk groups, including 5-Fluorouracil **(A)**, Sorafenib **(B)**, Tamoxifen **(C)**, Temozolomide **(D)**, Temsirolimus **(E)**, and Vinblastine **(F)**. **(G)** Sensitivity correlation analyses of the LRLs and potential drugs according to the CellMiner Database.

To further explore effective drugs for BC patients to guide precision treatment, we analyzed the sensitivity to common chemotherapeutic agents of the two risk groups. High-risk patients had higher IC50 of 5-Fluorouracil, Sorafenib, Tamoxifen, Temozolomide, Temsirolimus, and Vinblastine (Figures 9A–F), indicating they were more likely to be resistant to the above drugs. Furthermore, we explored the potential drugs targeted to the seven model genes with the CellMiner database. We finally acquired 16 gene-drug correlations, of which 11 correlations pointed to the USP30-AS1, and five correlations pointed to the C9orf163 (Figure 9G). C9orf163 expressed higher in the high-risk group, while C9orf163 expressed higher in the low-risk group. Ribavirin, Fulvestrant, SR16157, 8-Chloro-adenosine, and Methylprednisolone were positively related to C9orf163 so they might benefit high-risk patients. Conversely, the BRCA drug Ifosfamide was positively correlated with USP30-AS1; it might benefit low-risk patients (Figure 9G).

## Discussion

BC has the highest incidence rate among all cancers globally, which causes tens of thousands of female deaths yearly (Harbeck et al., 2019). BC is characterized by tumor heterogeneity at the molecular level of tumor cells and the tumor microenvironment (TME) (Baker et al., 2018; Sousa et al., 2019). Tumor heterogeneity complicates the aggressiveness and treatment of BC (Januškevičienė and Petrikaitė, 2019). Recent studies have revealed lactate’s diverse roles in the TME. Although cancer cells have a sufficient oxygen supply, they still use glucose and produce lactate excessively, which could cause acidosis, angiogenesis, and immunosuppression (Ho et al., 2019). In BC, lactate is correlated with resistance to PI3K inhibitors (Hillis and TOKER, 2020). In several cancers, lactate is essential in predicting prognosis, tumor microenvironment, and immune response (Mai et al., 2022; Sun et al., 2022; Xie et al., 2022). However, the prognostic value of lactate in BC remains largely unknown. This is the first study investigating the role of lactate in predicting prognosis, immune status, and therapeutic response in BC.

We first identified 196 differential expression LRLs for further study. We used the univariate Cox regression analysis, LASSO, and multivariate Cox regression analysis to construct the LRLPS. Survival analysis and the time-dependent ROC curves confirmed the prognostic value and reliability of the LRLPS. The AUC of the risk score was higher than other clinicopathological characteristics, indicating the highest prognostic performance of the LRLPS. Subsequent univariate and multivariate Cox regression analyses further indicated the independent prognostic predictability of the risk score. Stratified analysis showed that the LRLPS was suitable for patients in any clinical subgroup. The predictive ability of our signature was further explored by comparing it with various molecular signatures commonly used to predict OS in BC patients. Our signature displayed much higher AUCs than ferroptosis, necroptosis, pyroptosis, and immune-related signatures, which implied a stronger predictive ability, especially in predicting short-term survival status. Furthermore, the nomogram provided a powerful tool for clinicians to make decisions.

The GO/KEGG and GSEA indicated that the immune-related pathways differed between the two-risk groups. Previous research has demonstrated that lactate could regulate TMB. Through its ability to enhance the metabolic profile of the Treg and maintain acidity in the TME, lactate could enhance the immunosuppressive effect (Dastmalchi et al., 2021). Excessive lactate inhibits T-cell proliferation, such as Natural killer, dendritic, and CD8^+^ T cells (Haas et al., 2015; Certo et al., 2021; Grote et al., 2021). In addition, lactate could potentiate the anti-inflammatory effects by activating macrophages, promoting angiogenesis, tissue remodeling, and accelerating tumor growth and invasion (Certo et al., 2021). Hence, we further explore TIME through several algorithms. Tumor immune cell infiltration (TIICs) is a crucial component of the TIME. We calculated the levels of TIICs in BC with CIBERSORT. The high-risk group was enriched with immunosuppressive immune cells, such as macrophages M0 and M2, which were also critical members of EMT and cancer metastasis (Biswas and Mantovani, 2010; Qian and Pollard, 2010). Instead, CD4/8^+^ T cells, the vital factors in killing tumors and promoting immune response, were the main component in the low-risk group (Charoentong et al., 2017). According to the ESTIMATE analysis, the low-risk group had a higher immune score and stromal content while lower tumor purity than the high-risk group.

Immunotherapy has been a new treatment modality in BC, especially for metastatic BC (Adams et al., 2019). We further estimated the immunotherapy responses of the two risk groups. It is reported that ICIs antitumor relay on the CD8^+^ T cells, CD4^+^ T cells, and dendritic cells (Sato et al., 2018; Farhood et al., 2019). The immune cell infiltration levels were positively correlated with the responsiveness to ICIs (Karn et al., 2017; Kümpers et al., 2019). As an essential biomarker for predicting cancer immunotherapy (Patel and Kurzrock, 2015), the 27 ICPs expressed higher in the low-risk group. Therefore, we speculated that the low-risk group could respond better to immunotherapy and further verified the conclusion through TIDE and IPS analyses. All IPSs of CTLA4-/PD-1-, CTLA4 + /PD-1-, CTLA4-/PD-1 +, and CTLA4+/PD-1 + were higher in the low-risk group, indicted that the low-risk group had a better response to immunotherapy. Patients with high risk had the higher TMB in our study. Some research has indicated that TMB could act as a biomarker for predicting the response to ICIs (Lu et al., 2019; Shum et al., 2022). However, the predictive value varies among different cancers and might be insufficient in solid tumors (Xu et al., 2019). Thus, ICIs could benefit low-risk patients, while other immunotherapy might be appropriate for high-risk patients. These results indicated the significant differences in the degree of immune cell infiltration and immunotherapy response between the two risk groups identified by lactate-related signature.

Regarding the seven LRLs in our signature, some have been studied before in other cancers. USP30-AS1 is involved in autophagy, proliferation, and apoptosis in acute myeloid leukemia, glioblastoma, and cervical cancer (Chen et al., 2021; Wang et al., 2021; Zhou et al., 2022). In our study, USP30-AS1 was positively correlated with the antitumor immune cells and the classic ICPs. These results indicated the potential role of USP30-AS1 in TME. C9orf163 could develop the tumor microenvironment through cytokine and chemokine signaling and might act as a tumor suppressor in anaplastic gliomas and pancreatic cancer (Wang et al., 2016; Zhuang et al., 2020). Furthermore, we found that the expression of USP30-AS1 and C9orf163 were associated with BRCA1/2 status, indicating that they were involved in breast cancer development. However, more research is required to clarify the molecular mechanism of the seven LRLs in BC.

In treating BC, chemotherapeutic drugs could reduce tumor recurrence and be a primary treatment option for metastatic disease. However, chemo-resistance severely limited the clinical efficacy of chemotherapeutic drugs for BC patients (O’driscoll and Clynes, 2006). Thus, we assessed the BC patients’ response to chemotherapy with the IC50 value. The pRRophetic showed that low-risk patients were more sensitive to the common chemotherapy drugs, such as 5-Fluorouracil, Sorafenib, Tamoxifen, Temozolomide, Temsirolimus, and Vinblastine. Furthermore, we performed the CellMiner database to predict the candidate small-molecule compounds. The results indicated that Ribavirin, Fulvestrant, SR16157, 8-Chloro-adenosine, and Methylprednisolone might benefit high-risk patients. In combination, these discoveries may provide BC patients with suitable treatment options.

However, there were still a few limitations to our study. We used the TCGA dataset for all analyses since other databases lacked the needed LRLs data, including the Gene Expression Omnibus (GEO) and METABRIC databases, which prevented us from verifying the results. Therefore, it is better to validate in a prospective cohort. Secondly, further studies on the biological functions of the seven LRLs are needed to be performed *in vivo* and *in vitro*.

## Conclusion

Altogether, this study identified a novel lactate-related lncRNAs prognostic signature for BC patients, which could predict the prognosis and immune infiltration. The LRLPS also provided an effective method for personalized risk estimation and assessment of treatment response to immunotherapy and chemotherapy, which may be clinically helpful. Finally, the seven LRLs could become potential treatment targets for BC.

## Data Availability

The data can be found here: https://portal.gdc.cancer.gov/repository.
