# Supplementary material for: A lactate-related LncRNA model for predicting prognosis, immune landscape and therapeutic response in breast cancer
Source: Front Genet. 2022 Oct 5;13:956246. doi: 10.3389/fgene.2022.956246 (PMC9579365; doi:10.3389/fgene.2022.956246)
Supplement: Supplementary file 1 [file Table1.DOCX]

| n | level | Entire cohort n=916 | Taining cohort n=458 | Test cohort n=458 | p |
| --- | --- | --- | --- | --- | --- |
| age (%) | <=60 | 507 ( 55.3) | 255 ( 55.7) | 252 ( 55.0) | 0.98 |
|  | >60 | 409 ( 44.7) | 203 ( 44.3) | 206 ( 45.0) |  |
| Stage (%) |  | 20 ( 2.2) | 14 ( 3.1) | 6 ( 1.3) | 0.732 |
|  | I | 169 ( 18.4) | 79 ( 17.2) | 90 ( 19.7) |  |
|  | II | 506 ( 55.2) | 261 ( 57.0) | 245 ( 53.5) |  |
|  | III | 203 ( 22.2) | 96 ( 21.0) | 107 ( 23.4) |  |
|  | IV | 18 ( 2.0) | 8 ( 1.7) | 10 ( 2.2) |  |
| T (%) | T1 | 254 ( 28.0) | 120 ( 26.5) | 134 ( 29.5) | 0.861 |
|  | T2 | 500 ( 55.1) | 261 ( 57.7) | 239 ( 52.5) |  |
|  | T3 | 121 ( 13.3) | 58 ( 12.8) | 63 ( 13.8) |  |
|  | T4 | 29 ( 3.2) | 11 ( 2.4) | 18 ( 4.0) |  |
|  | TX | 3 ( 0.3) | 2 ( 0.4) | 1 ( 0.2) |  |
| N (%) | N0 | 426 ( 47.0) | 198 ( 43.8) | 228 ( 50.1) | 0.508 |
|  | N1 | 308 ( 34.0) | 166 ( 36.7) | 142 ( 31.2) |  |
|  | N2 | 93 ( 10.3) | 42 ( 9.3) | 51 ( 11.2) |  |
|  | N3 | 65 ( 7.2) | 36 ( 8.0) | 29 ( 6.4) |  |
|  | NX | 15 ( 1.7) | 10 ( 2.2) | 5 ( 1.1) |  |
| M (%) | M0 | 746 ( 82.3) | 368 ( 81.4) | 378 ( 83.3) | 0.901 |
|  | M1 | 18 ( 2.0) | 8 ( 1.8) | 10 ( 2.2) |  |
|  | MX | 142 ( 15.7) | 76 ( 16.8) | 66 ( 14.5) |  |
| ER (%) | NA | 18 ( 2.0) | 9 ( 2.0) | 9 ( 2.0) | 0.65 |
|  | Indeterminate | 2 ( 0.2) | 2 ( 0.4) | 0 ( 0.0) |  |
|  | Negative | 200 ( 21.8) | 109 ( 23.8) | 91 ( 19.9) |  |
|  | Positive | 696 ( 76.0) | 338 ( 73.8) | 358 ( 78.2) |  |
| PR (%) | NA | 18 ( 2.0) | 9 ( 2.0) | 9 ( 2.0) | 0.742 |
|  | Indeterminate | 3 ( 0.3) | 3 ( 0.7) | 0 ( 0.0) |  |
|  | Negative | 289 ( 31.6) | 149 ( 32.5) | 140 ( 30.6) |  |
|  | Positive | 606 ( 66.2) | 297 ( 64.8) | 309 ( 67.5) |  |
| HER2 (%) | NA | 140 ( 15.3) | 75 ( 16.4) | 65 ( 14.2) | 0.998 |
|  | Equivocal | 159 ( 17.4) | 77 ( 16.8) | 82 ( 17.9) |  |
|  | Indeterminate | 12 ( 1.3) | 6 ( 1.3) | 6 ( 1.3) |  |
|  | Negative | 484 ( 52.8) | 238 ( 52.0) | 246 ( 53.7) |  |
|  | Positive | 121 ( 13.2) | 62 ( 13.5) | 59 ( 12.9) |  |

Supplementary table 1: The clinical features of the patients in the training, test and entire cohorts.

Supplementary table 2: The 284 lactate-related genes.

| gene |
| --- |
| ACTN3 |
| HAGH |
| HIF1A |
| LDHA |
| LDHAL6B |
| LDHB |
| LDHC |
| LDHD |
| MIR210 |
| PARK7 |
| PER2 |
| PFKFB2 |
| PNKD |
| SLC25A12 |
| TIGAR |
| TP53 |
| AARS2 |
| ACAD9 |
| ACAT1 |
| ACAT2 |
| ADAMTS13 |
| AGK |
| AIFM1 |
| ATAD3A |
| ATPAF2 |
| BCS1L |
| C1QBP |
| CA5A |
| CARS2 |
| CHCHD10 |
| CLPB |
| COQ2 |
| COQ4 |
| COQ8A |
| COQ9 |
| COX10 |
| COX14 |
| COX15 |
| COX20 |
| COX4I1 |
| COX5A |
| COX6A2 |
| COX6B1 |
| COX8A |
| CYC1 |
| CYP27A1 |
| DARS2 |
| DGUOK |
| DLD |
| DNAJC19 |
| DNM1L |
| EARS2 |
| ECHS1 |
| FARS2 |
| FASTKD2 |
| FBXL4 |
| FDX2 |
| FOXRED1 |
| GFM1 |
| GFM2 |
| GOT2 |
| GTPBP3 |
| GYS2 |
| HIBCH |
| HMGCL |
| HPDL |
| HS6ST2 |
| HSD17B10 |
| HTRA2 |
| ISCA1 |
| ISCU |
| KARS1 |
| LIAS |
| LIPT1 |
| LIPT2 |
| LONP1 |
| LRPPRC |
| LYRM7 |
| MDH2 |
| MECP2 |
| MICOS13 |
| MIPEP |
| MPC1 |
| MRPL12 |
| MRPL3 |
| MRPL44 |
| MRPS14 |
| MRPS16 |
| MRPS22 |
| MRPS28 |
| MRPS34 |
| MT-ATP6 |
| MT-CO1 |
| MT-CO2 |
| MT-CO3 |
| MT-ND1 |
| MT-ND2 |
| MT-ND3 |
| MT-ND4 |
| MT-ND5 |
| MT-ND6 |
| MT-TF |
| MT-TH |
| MT-TI |
| MT-TK |
| MT-TL1 |
| MT-TL2 |
| MT-TN |
| MT-TP |
| MT-TQ |
| MT-TS1 |
| MT-TS2 |
| MT-TV |
| MT-TW |
| MTFMT |
| MTO1 |
| MTRFR |
| NARS2 |
| NAXE |
| NDUFA1 |
| NDUFA10 |
| NDUFA11 |
| NDUFA12 |
| NDUFA13 |
| NDUFA2 |
| NDUFA4 |
| NDUFA8 |
| NDUFA9 |
| NDUFAF1 |
| NDUFAF2 |
| NDUFAF3 |
| NDUFAF5 |
| NDUFAF6 |
| NDUFB8 |
| NDUFB9 |
| NDUFC2 |
| NDUFS1 |
| NDUFS2 |
| NDUFS3 |
| NDUFS4 |
| NDUFS7 |
| NDUFS8 |
| NDUFV1 |
| NDUFV2 |
| NFS1 |
| NGLY1 |
| OCRL |
| OGDH |
| PC |
| PDHA1 |
| PDHX |
| PDP1 |
| PDSS1 |
| PDSS2 |
| PET100 |
| PET117 |
| PHKG2 |
| PITRM1 |
| PMPCB |
| PNPLA8 |
| PNPO |
| PNPT1 |
| POLG |
| POLG2 |
| PUS1 |
| PYGL |
| RARS1 |
| RARS2 |
| RMND1 |
| RNASEH1 |
| RRM2B |
| SCO1 |
| SCO2 |
| SDHA |
| SDHB |
| SERAC1 |
| SFXN4 |
| SLC19A3 |
| SLC25A10 |
| SLC25A26 |
| SLC25A3 |
| SLC25A4 |
| SLC25A42 |
| SLC7A7 |
| SOD1 |
| SUCLG1 |
| SURF1 |
| SYNJ1 |
| TACO1 |
| TANGO2 |
| TARS2 |
| TIMM22 |
| TIMM50 |
| TK2 |
| TMEM126B |
| TMEM70 |
| TRMT10C |
| TRMT5 |
| TRMU |
| TSFM |
| TUFM |
| TWNK |
| TXN2 |
| UQCC3 |
| UQCRB |
| UQCRC2 |
| UQCRQ |
| WARS2 |
| YARS1 |
| YARS2 |
| ALDH7A1 |
| ALDOB |
| ATP5F1D |
| ATP5F1E |
| BCKDHA |
| BCKDHB |
| BOLA3 |
| CAMKMT |
| COA6 |
| COA8 |
| COX16 |
| DBT |
| DLAT |
| ELAC2 |
| ETHE1 |
| FBP1 |
| FH |
| G6PC1 |
| GATB |
| GATC |
| GFER |
| HADH |
| HADHA |
| HADHB |
| IBA57 |
| LARS1 |
| LARS2 |
| LYRM4 |
| MLYCD |
| MPV17 |
| MRPS7 |
| MT-CYB |
| MT-TC |
| MT-TE |
| MT-TT |
| NADK2 |
| NDUFA6 |
| NDUFAF4 |
| NDUFAF8 |
| NDUFB10 |
| NDUFB11 |
| NDUFB3 |
| NDUFS6 |
| NFU1 |
| NUBPL |
| PCCA |
| PCCB |
| PCK1 |
| PDHB |
| PHKA2 |
| PLPBP |
| PMPCA |
| PPM1B |
| PREPL |
| QRSL1 |
| RYR1 |
| SDHD |
| SLC13A5 |
| SLC25A13 |
| SLC25A19 |
| SLC37A4 |
| SLC3A1 |
| SQOR |
| SUCLA2 |
| TAFAZZIN |
| TIMMDC1 |
| TKFC |
| TPK1 |
| TRNT1 |
| TYMP |
| UQCRFS1 |
| USP18 |
| GATM |
| SLC25A21 |

Supplementary table 3: The 7 lncRNAs were used to construct the risk mode.

|  | coef | exp(coef) | se(coef) | z | Pr(>\|z\|) |
| --- | --- | --- | --- | --- | --- |
| C9orf163 | 1.915272666 | 6.788789617 | 0.57292615 | 3.342966027 | 0.00082888 |
| RP1-28O10.1 | -0.677100153 | 0.508088237 | 0.39158774 | -1.72911478 | 0.08378855 |
| RP11-496I9.1 | -0.503780886 | 0.604241766 | 0.30548504 | -1.64911803 | 0.09912346 |
| CTD-3065J16.9 | 1.048467864 | 2.853276161 | 0.27721459 | 3.782152598 | 0.00015548 |
| USP30-AS1 | -0.692769124 | 0.500189064 | 0.33766763 | -2.05163026 | 0.04020561 |
| LINC01569 | -0.835154753 | 0.433807343 | 0.39172011 | -2.13201908 | 0.03300528 |
| RP11-707G18.1 | -1.077081426 | 0.340588108 | 0.48811154 | -2.20662971 | 0.02733994 |

Supplementary Figure


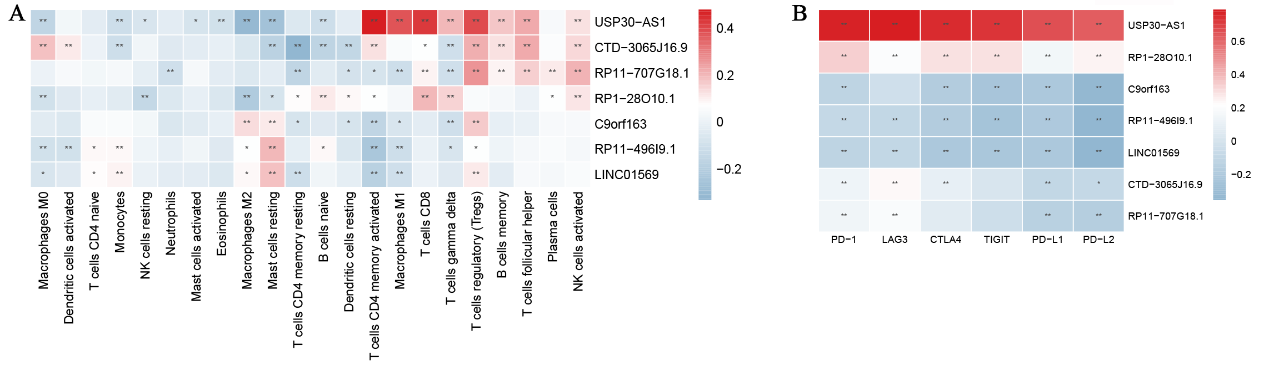


Supplementary Figure 1 The relationship between immune cells and the seven LRLs in our signature.

(A) The correlation between the immune cell infiltrates and the model LRLs. (B) The correlation between ICIs and the model LRLs.
